# Breast MRI in the Assessment of Internal Thoracic Lymph Nodes: Morphodynamic and Functional Criteria for Detection of Metastatic Lymph Nodes

**DOI:** 10.7759/cureus.101644

**Published:** 2026-01-15

**Authors:** Mirjan M Nadrljanski, Luka J Raspopovic, Dejan Dimitrijevic, Iva B Krusac, Marko N Mihajlovic, Andjela Djajic

**Affiliations:** 1 Department of Radiology, Faculty of Medicine, University of Belgrade, Belgrade, SRB; 2 Department of Diagnostic Radiology, Institute of Oncology and Radiology of Serbia, Belgrade, SRB

**Keywords:** adc, dwi, internal thoracic artery lymph nodes, long axis, mri, short axis, volume

## Abstract

Introduction: Metastasis to internal thoracic artery lymph nodes (ITLNs) occurs in a substantial proportion of patients with breast carcinoma and is associated with adverse prognosis. Despite its clinical importance, standardized imaging criteria for ITLN assessment are lacking. This study aimed to evaluate morphological and functional MRI features that differentiate metastatic from physiological ITLNs in breast cancer patients.

Methods: In this retrospective single-center study, 34 patients with histologically confirmed invasive breast carcinoma and detectable ITLNs on breast MRI were included. Sixteen patients had metastatic ITLNs (confirmed histologically or by fluorodeoxyglucose positron emission tomography), while 18 patients had non-metastatic ITLNs with imaging stability on follow-up. All patients underwent breast MRI, including dynamic contrast-enhanced (DCE)-MRI and diffusion-weighted imaging (DWI). Morphological parameters (short axis, long axis, short-to-long axis ratio, volume, surface area, and compactness) and functional parameters (apparent diffusion coefficient (ADC) and positive enhancement integral (PEI)) were analyzed and compared between groups.

Results: Metastatic ITLNs demonstrated significantly larger short and long axis diameters, greater volume and surface area, lower compactness, and significantly lower ADC values compared with non-metastatic ITLNs (all p < 0.0001). The short-to-long axis ratio and PEI showed smaller differences between groups and were less discriminatory. The ADC values were significantly lower in metastatic ITLNs, reflecting increased cellularity. Cut-off values for multiple parameters demonstrated high diagnostic performance, with sensitivities and specificities exceeding 90%.

Conclusions: Morphological MRI parameters, particularly lymph node size, volume, surface area, compactness, and ADC values, provide reliable criteria for distinguishing metastatic from physiological ITLNs in breast cancer patients. Incorporation of these morpho-functional MRI features into routine breast MRI assessment may improve diagnostic accuracy and staging. Larger prospective multicenter studies are warranted to validate these findings and establish standardized imaging protocols.

## Introduction

Internal thoracic artery lymph nodes (ITLNs), also known as internal mammary lymph nodes, form a bilateral lymphatic chain located parasternally along the internal thoracic artery and vein, extending from the first to the sixth anterior intercostal space. The superior ITLNs of both sides may communicate through the retromanubrial lymph nodes [1,2]. Considerable individual variability exists in ITLN anatomy, with the second and third intercostal spaces most commonly containing at least one node and the highest number typically observed in the third intercostal space [3]. Physiological ITLNs are generally small, measuring approximately 1 mm to 5 mm [2]. On MRI, the reported average ITLN size ranges from 2 mm to 9 mm, with a mean diameter of approximately 4.5 ± 1.59 mm [4]. The internal thoracic lymphatic trunk on the right typically drains into lymph nodes at the brachiocephalic angle and communicates with lymphatic pathways of the lung and esophagus, whereas on the left it terminates at the superior phrenic lymph nodes, joining drainage routes from Botallo’s lymph nodes and communicating with left pulmonary lymphatics [1,5]. Ultimately, both lymphatic trunks empty into the venous system [2].

Metastatic involvement of ITLNs occurs in approximately 16% to 52% of patients with breast carcinoma, reflecting their role as a secondary lymphatic drainage pathway [6,7]. The presence of ITLN metastases is associated with an unfavorable prognosis and disease upstaging [8]. Routine surgical sampling of ITLNs is uncommon in contemporary breast cancer management, as radical mastectomy has largely been replaced by less aggressive surgical approaches. Nevertheless, radiotherapy targeting ITLNs and systemic chemotherapy have been shown to reduce recurrence rates and improve survival in selected patients [6-9]. Metastatic involvement of ITLNs may be underestimated in clinical practice, particularly when axillary lymph node status dominates staging considerations, prompting renewed interest in sentinel lymph node biopsy of ITLNs for more accurate nodal assessment [10]. Incidental detection of ITLNs on imaging poses a diagnostic challenge, as not all visualized nodes are metastatic, underscoring the need for reliable imaging criteria to guide clinical decision-making.

Breast MRI has demonstrated high sensitivity (>90%) for the detection of ITLNs and for differentiating physiological from metastatic nodes based on size and morphological features [6,11]. In addition to morphologic assessment, functional imaging techniques provide complementary information. Both fluorodeoxyglucose (FEG) PET/CT and breast MRI may be used in a complementary manner to improve ITLN characterization and provide prognostic information [12].

This study aimed to evaluate whether selected morphological parameters, namely short axis, long axis, short-to-long axis (S/L) ratio, volume, surface area, and compactness, as well as functional MRI parameters, including the apparent diffusion coefficient (ADC) and positive enhancement integral (PEI) derived from dynamic contrast-enhanced (DCE)-MRI, can reliably differentiate metastatic from physiological ITLNs in patients with breast cancer. Secondary objectives included determining optimal cut-off values for individual parameters and an exploratory assessment of correlations between morphological and functional metrics.

An abstract of this research was previously presented at the European Congress of Radiology, organized by the European Society of Radiology, Vienna, AUT (February 28-March 4, 2018).

## Materials and methods

Study design and population

This retrospective, single-center study included female patients with histologically confirmed invasive breast carcinoma and detectable ITLNs on breast MRI. Following screening of 86 patients identified through the institutional hospital information system, 34 patients met the inclusion criteria and were enrolled in the analysis.

Patients were divided into two groups. The metastatic ITLN group (N1 = 16) consisted of patients with ITLNs identified on breast MRI that were confirmed as metastatic either histologically or by fluorodeoxyglucose positron emission tomography/computed tomography (FDG PET/CT) positivity, defined as focal FDG uptake above background mediastinal activity corresponding to a morphologically identifiable ITLN in patients with histologically confirmed invasive breast carcinoma.

The control group (N2 = 18) included age-matched breast cancer patients with detectable ITLNs that were considered non-metastatic based on stability on serial MRI examinations for at least one year and/or negative FDG PET/CT findings.

Exclusion criteria were the presence of distant metastases, unilateral or bilateral silicone breast implants, prior thoracic surgical or interventional procedures, significant motion or susceptibility artifacts on T1-weighted (T1W) or T2-weighted (T2W) images, and the absence of both axial and coronal T2W sequences. Image acquisition and analysis were performed over an eight-year period (June 2016 to June 2024). The study was approved by the Institutional Review Board of the Institute of Oncology and Radiology of Serbia (approval no. 2044/12052010).

MRI acquisition protocol

All breast MRI examinations were performed using a 1.5-T MRI system (Magnetom Avanto or Magnetom Avanto Fit; Siemens Healthineers, Erlangen, DEU) with a dedicated bilateral breast coil. The standardized diagnostic protocol included T2W, T1W, diffusion-weighted imaging (DWI), and DCE-MRI sequences. Slice thickness was 2 mm for all sequences. The imaging parameters for each sequence are summarized in Table 1. The DWI was acquired using b-values of 50 and 850 s/mm², and ADC maps were generated automatically by the scanner software. For DCE-MRI, one pre-contrast and five post-contrast T1W fast low-angle shot (FLASH) 3D series were acquired at intervals of 1 minute and 23 seconds. The contrast agent gadobutrol (1.0 mmol/mL; Gadovist, Bayer Pharma, Berlin, DEU) was administered intravenously at a dose of 0.1 mmol/kg body weight using an automatic injector at a rate of 2 mL/s, followed by a 20 mL saline flush.

**Table 1:** Breast DCE-MRI protocol details *One pre- and five post-contrast series (every 1 minute 23 seconds) DCE: Dynamic contrast-enhanced, TE: Echo time, TR: Repetition time, FOV: Field of view, T2W TIRM: Turbo inversion recovery magnitude, TSE: Turbo spin echo, T1W: T1-weighted, T2W: T2-weighted, FLASH 3D: Fast low-angle shot 3D, DWI: Diffusion weighted imaging

Parameter	T2W turbo inversion recovery magnitude (TIRM)	T2W-turbo spin echo (TSE) - axial/coronal	T1W-TSE	T1W FLASH 3D*	DWI (axial, b50, b850 s/mm2)
Echo time (TE)/Repetition time (TR)	60 ms / 7690 ms	70 ms / 5900 ms	12 ms / 920 ms	4.8 ms / 9.1 ms	75 ms / 1800 ms
Flip angle	150°	180°	90°	25°	-
Field of view (FOV)	340 x 340 mm				-
Image matrix	320 x 256	384 x 319	320 x 234	576 x 564	-

Image analysis

Morphological and functional analyses were performed on a dedicated workstation (Leonardo, Siemens Healthineers, Erlangen, DEU) using Syngo (Siemens Healthineers) and OsiriX (Pixmeo, Geneva, CHE) software. For each ITLN, the following morphological parameters were measured: short axis, long axis, S/L ratio, volume, surface area, and compactness. Measurements were obtained on T2W axial and coronal images, where lymph nodes were best visualized. Volume and surface area were calculated assuming an oblate ellipsoid geometry. Compactness was defined as the ratio of surface area to volume, expressed in units of inverse length (mm⁻¹), and used as a relative morphometric descriptor reflecting lymph node shape irregularity rather than as a dimensionless parameter.

Functional assessment included ADC values derived from DWI and positive enhancement integral (PEI) values obtained from DCE-MRI. The ADC measurements were performed by placing a circular region of interest (ROI) over the area of highest signal intensity on high b-value (b = 850 s/mm²) DWI images, with corresponding verification on ADC maps. The ROI size was standardized to approximately 8 ± 2 mm², and areas affected by T2 shine-through or high signal intensity on T2W images were avoided. The PEI color maps were generated on a pixel-by-pixel basis using Syngo software. The ROIs for PEI analysis were placed over the most enhancing portion of the lymph node on PEI maps, with circular ROI sizes of approximately 8 ± 2 pixels.

All measurements were performed by two radiologists with experience in breast MRI, working in consensus. They were aware of the diagnosis of primary breast cancer but were blinded to the metastatic status of ITLNs at the time of image analysis. Formal inter-observer variability analysis was not performed, which is acknowledged as a limitation of the study.

Statistical analysis

Two diameters were defined for each lymph node: short axis ((S), mm) and long axis ((L), mm), which also served for the calculation of the S/L ratio and the surface area (SA) of the ellipsoid, volume (V) and compactness (C), based on the following formulae [13, 14]:

Surface area of an oblate ellipsoid \begin{equation} SA = \frac{2 \pi \left[ S^2 + (S \times L \times \alpha) \right]}{\sin \alpha} \end{equation}

Alpha angle \begin{equation} \alpha = \arccos \left( \frac{S}{L} \right) \end{equation}

Volume of an oblate ellipsoid \begin{equation} V = \frac{4}{3} \pi (S^2 \times L) \quad [\mathrm{mm}^3] \end{equation}

Compactness (surface-to-volume ratio) \begin{equation} C = \frac{SA}{V} \end{equation}

Statistical analysis was performed using the open-access BiostaTGV software (Institut Pierre Louis, UMR S 1136, Inserm and Sorbonne University, Paris, FRA). Group comparisons were conducted using the two-tailed Mann-Whitney U test. A more stringent significance threshold of α = 0.01 was applied in order to reduce the risk of type I error arising from multiple simultaneous comparisons across several morphologic and functional parameters in a relatively small study cohort. Spearman's correlation test and the assessment of sensitivity, specificity, and Youden's index [15] for cut-off values between physiological and metastatic ITLNs were performed [16].

## Results

Patient characteristics

The mean age of the included female patients was 54.5 ± 10.5 years. There was no statistically significant difference in age between patients with metastatic ITLNs (N1: 55.94 ± 9.58 years) and those with non-metastatic ITLNs (N2: 53.22 ± 11.59 years; p = 0.435). Breast tumor size did not differ significantly between the two groups (N1: 2.39 ± 0.43 cm vs. N2: 2.37 ± 0.32 cm; p = 0.960).

Morphological MRI parameters

Metastatic ITLNs demonstrated significantly larger short and long axis diameters compared with non-metastatic nodes (both p < 0.0001). The long axis diameter exceeded 11 mm in all metastatic lymph nodes. The short-to-long axis ratio was higher in metastatic nodes; however, this difference was less pronounced (p = 0.002). Three-dimensional morphological parameters showed marked differences between the groups. Metastatic ITLNs exhibited significantly greater volume and surface area, as well as significantly lower compactness, compared with non-metastatic nodes (all p < 0.0001).

Functional MRI parameters and their comparison with morphological parameters

The ADC values were significantly lower in metastatic ITLNs compared with non-metastatic nodes (p < 0.0001). The PEI values were higher in metastatic lymph nodes, although the difference was less pronounced (p = 0.002). All assessed morphological and functional parameters differed significantly between metastatic and non-metastatic ITLNs (Table 2). Except for the S/L ratio and PEI, all parameters demonstrated highly significant differences between the two groups. Mann-Whitney U values indicated strong discrimination between metastatic and non-metastatic lymph nodes for size, volume, compactness, and ADC-based parameters, whereas the S/L ratio and PEI demonstrated lower discriminatory strength with partial overlap between groups.

**Table 2:** Morphologic and functional parameters in metastatic and non-metastatic ITLNs Significance between N1 and N2 was tested with a two-tailed Mann-Whitney test with significance at 0.01 for the p-value. S/L ratio: Short to long axis ratio, ADC: Apparent diffusion coefficient, PEI: Positive enhancement integral, LN: Lymph node, ITLNs: Internal thoracic artery lymph nodes

Parameters	Metastatic LNs (N1=16)	Non-metastatic LNs (N2=18)	U-value	p-value
Short axis (mm)	7.68+/-0.87	4.50+/-0.86	287.0	p<0.0001
Long axis (mm)	13.00+/-1.75	8.62+/-1.61	277.5	p<0.0001
S/L ratio (x+/-SD)	0.59+/-0.08	0.53+/-0.09	214.0	p=0.002
Volume (mm^3^)	410.31+/-108.53	98.26+/-48.71	286.5	p<0.0001
Surface area (mm^2)^	277.28+/-50.69	107.19+/-35.43	286.5	p<0.0001
Compactness (mm^-1)^	0.69+/-0.07	1.19+/-0.22	0.5	p<0.0001
ADC (mm^2^/s x 10-3)	0.58+/-0.06	0.80+/-0.05	1.0	p<0.0001
PEI (x+/-SD )	636.97+/-74.50	530.44+/-68.85	126.5	p=0.002

Correlation analysis and diagnostic cut-off values

Spearman’s rank correlation analysis demonstrated a moderate inverse correlation between short axis diameter and ADC values (R = −0.429). However, this correlation did not reach statistical significance (p = 0.09). Cut-off values were established for each parameter to differentiate metastatic from physiological ITLNs (Table 3). Sensitivity and specificity exceeded 90% for all evaluated parameters. Short axis (> 6.5 mm), long axis (> 11.5 mm), and PEI (> 550) demonstrated the highest Youden’s index (0.85), indicating the best overall diagnostic performance.

**Table 3:** Cut-off values for all parameters discriminating physiologic from metastatic ITLNs with sensitivity and specificity of at least 90% ITLNs: Internal thoracic artery lymph nodes, S: Short axis, L: Long axis, S/L: Short-to-long axis ratio, V: Volume, SA: Surface area, C: Compactness, ADC: Apparent diffusion coefficient, PEI: Positive enhancement integral

Parameter	Cut-off	Sensitivity	Specificity	Youden's index
Short axis	S > 6.5 mm	90%	95%	0.85
Long axis	L > 11.5 mm	91%	94%	0.85
S/L ratio	S/L < 0.65	89%	93%	0.82
Volume	V > 140 mm³	90%	93%	0.83
Surface area	SA > 140 mm²	91%	92%	0.83
Compactness	C < 0.85 mm^-1^	92%	91%	0.83
ADC	ADC < 0.7 × 10⁻³ mm²/s	91%	92%	0.83
PEI	PEI > 550	90%	95%	0.85

Representative case

**Figure 1:**
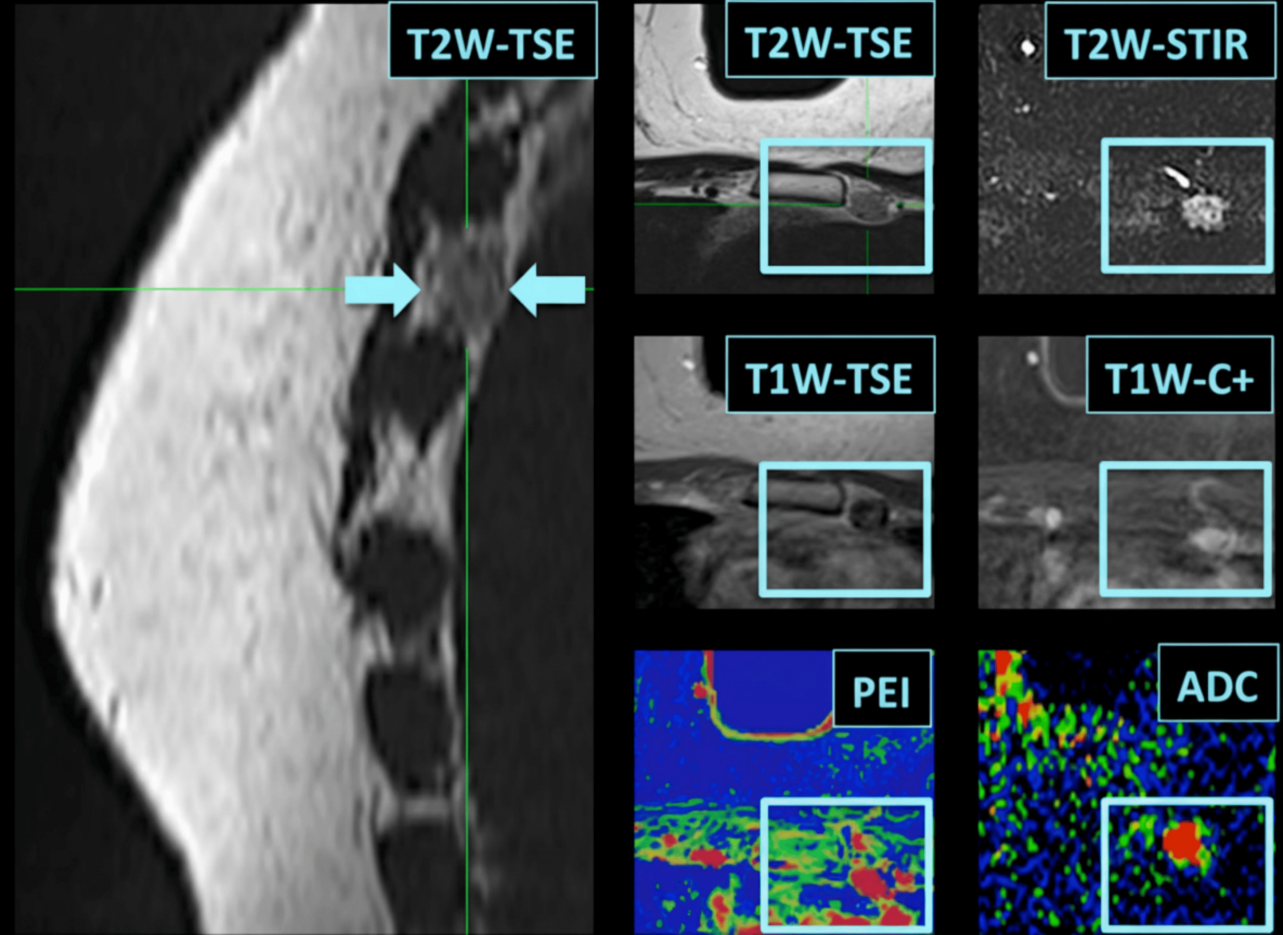
Breast MRI of a 66-year-old patient with histologically confirmed metastatic ITLN The lymph node is visualized on sagittal T2W TSE images (arrows) and axial T2W short tau inversion recovery (STIR), T2W TSE, T1W TSE, contrast-enhanced T1W images, and corresponding ADC and PEI maps. ITLN: Internal thoracic lymph node, TSE: Turbo spin echo, STIR: Short tau inversion recovery, C+: Contrast enhanced, ADC: Apparent diffusion coefficient, PEI: Positive enhancement integral, T1W: T1-weighted, T2W: T2-weighted

Figure 1 presents a representative case of a metastatic ITLN identified on breast MRI, demonstrating increased lymph node size and reduced ADC value, with histopathological confirmation.

The ratio between the S/L used in imaging to assess the overall shape of the lymph node did not prove highly significant in our study. The deviation from an ovoid shape to a spherical shape (S/L ratio closer to 1) is typically associated with metastatic lymph nodes. When volume and surface area are considered, more pronounced morphological differences between benign and malignant nodes are observed. Metastatic lymph nodes are significantly larger in volume and surface area compared to benign ones. Volume reflects the overall size, while surface area provides information on how much contact area the lymph node has with its surroundings, which can be influenced by irregular growth patterns. Surface area and volume are more indicative of malignant transformation compared to simple size measurements (short axis and long axis).

Compactness is lower in metastatic nodes due to irregularities in their shape. Metastatic lymph nodes tend to be less compact than benign ones because of their non-ovoid, more distorted shapes. As mentioned, the ratio between the short axis and the long axis was not highly significant in our study. However, this ratio can still offer insights into the shape of the lymph node. In benign nodes, the S/L ratio tends to be closer to 0.5-0.6, which indicates an elongated ovoid shape. Metastatic nodes, on the other hand, may have a higher S/L ratio (closer to 1), indicating that the nodes are more spherical as the lymph nodes grow and distort the original shape.

According to the data regarding the correlation (Spearman’s rank) between the parameters within the data subsets, only moderate correlation was reported between short axis and ADC (R=-0.429, with p=0.09), which was not considered significant. Sensitivity, specificity, and Youden's index were calculated for all suggested cut-off values for discrimination between physiologic and metastatic ITLNs based on each parameter assessed on routine breast MRI studies, as presented in Table 3. All parameters in Table 3 demonstrated sensitivity and specificity values exceeding 90%, with specificity reaching up to 95% for surface area and PEI. Youden’s index values ranged from 0.82 to 0.85. Short axis, long axis, and PEI yielded the highest Youden’s index (0.85), while S/L ratio, volume, surface area, compactness, and ADC demonstrated Youden’s index values between 0.82 and 0.83.

## Discussion

This study demonstrates that a combination of morphologic and functional MRI parameters enables reliable differentiation between metastatic and physiological ITLNs in patients with breast cancer. The practical implication of these findings lies in the potential routine use of breast MRI-derived parameters for ITLN assessment, given the known prognostic significance of ITLN metastases and their role in disease upstaging to clinical stage III.

The presence of metastatic ITLNs has been associated with specific clinical and tumor-related factors, including medially located and deeply seated primary tumors, younger patient age, axillary lymph node involvement, higher nuclear grade, and the triple-negative breast cancer subtype [17]. Accurate preoperative identification of ITLN involvement may therefore contribute to improved staging and treatment planning. Other imaging modalities, such as ultrasound, have limited sensitivity for ITLN visualization, as physiologic ITLNs are frequently not detectable [18]. From a technical standpoint, the addition of a coronal T2W or T1W TSE sequence centered on the sternum may improve ITLN visualization with minimal extension of examination time. This adjustment may facilitate more consistent identification and assessment of ITLNs across routine breast MRI studies.

Consistent with previous reports, metastatic ITLNs in this study demonstrated significantly larger long-axis diameters, with all metastatic nodes exceeding 11 mm [6,11,17]. However, lymph node size alone remains an imperfect discriminator, as physiological ITLNs may also reach relatively large dimensions [19]. Prior studies have proposed various size-based cut-off values, often based on limited cohorts and purely morphologic criteria [17-19]. In contrast, the present study incorporated multiple morphologic and functional parameters, which collectively improved discriminatory performance.

Short-axis diameter was significantly greater in metastatic ITLNs, supporting its role as a useful morphologic indicator. Although a moderate inverse correlation between short-axis diameter and ADC values was observed, this relationship did not reach statistical significance. Compared with qualitative diffusion-weighted imaging approaches used in earlier studies, the quantitative ADC-based assessment applied in this study yielded comparable diagnostic performance while avoiding variability related to binary diffusion restriction assessment. The S/L ratio demonstrated statistically significant differences between groups; however, its discriminatory value was lower compared with other parameters [20]. This finding may be related to the relatively limited size range of ITLNs in the study population.

Three-dimensional morphologic parameters, particularly lymph node volume and surface area, showed strong discriminatory power. Metastatic ITLNs exhibited substantially greater volume and surface area and lower compactness compared with physiologic nodes. These parameters may better reflect global nodal enlargement and shape distortion than linear measurements alone. Although 3D morphometric parameters demonstrated strong discriminatory performance, this study did not include multivariate modeling; therefore, independent incremental diagnostic value beyond size-based criteria cannot be conclusively established.

Among functional parameters, ADC values provided robust differentiation between metastatic and physiologic ITLNs, with minimal overlap between groups. In contrast, PEI demonstrated overlapping values between metastatic and non-metastatic nodes, potentially reflecting technical challenges related to region-of-interest placement in small lymph nodes.

It should be noted that the true incidence of ITLN metastases may be underestimated in clinical practice. A systematic imaging approach incorporating optimized MRI protocols and multiparametric analysis may improve the detection and characterization of ITLNs. Differential diagnoses for ITLN enlargement include inflammatory conditions, silicone implant-related changes, and metastatic involvement from non-breast malignancies, which should be considered during interpretation [18].

This study has several limitations. Its retrospective, single-center design and relatively small sample size may limit generalizability and increase the risk of cut-off overfitting. In addition, FDG PET/CT was used as a surrogate reference standard for nodal metastasis in a subset of patients, which may introduce verification bias. Histopathological confirmation was not available for all lymph nodes in the control group. Furthermore, formal inter-observer variability analysis was not performed. 

The results of this study support the inclusion of ITLN assessment in routine breast cancer staging using multiparametric MRI. Accurate identification of ITLN metastases may have important implications for staging, prognosis, and management strategies in breast cancer patients.

## Conclusions

Breast MRI enables reliable differentiation between metastatic and physiological ITLNs using a combination of morphologic and functional imaging parameters. In this study, lymph node size, 3D morphologic descriptors, and quantitative diffusion metrics demonstrated significant discriminatory value. Systematic assessment of ITLNs on routine breast MRI may support more accurate staging and treatment planning. Further prospective, multicenter studies are required to validate the proposed cut-off values and to establish standardized imaging criteria.
